# Wearable technology use for the analysis and monitoring of functions related to feeding and communication

**DOI:** 10.1590/2317-1782/20212021278en

**Published:** 2022-07-22

**Authors:** Bianca Oliveira Ismael da Costa, Alana Moura Xavier Dantas, Liliane dos Santos Machado, Hilton Justino da Silva, Leandro Pernambuco, Leonardo Wanderley Lopes

**Affiliations:** 1 Programa de Pós-graduação em Modelos de Decisão e Saúde, Universidade Federal da Paraíba – UFPB - João Pessoa (PB), Brasil.; 2 Programa de Pós-graduação em Odontologia, Cidade Universitária, Universidade Federal de Pernambuco – UFPE - Recife (PE), Brasil.

**Keywords:** Mastication, Deglutition, Voice, Wearables, Biomedical Technology

Dear editors,

Wearable devices and systems are contemporary alternatives to overcome challenges in the analysis and monitoring of functions related to feeding and communication. The objective of this letter is to comment on this scenario in the fields of mastication, swallowing, and voice.

Feeding and communication are indispensable to human survival, have social and emotional aspects in common, and are both dependent on the physiology of the head and neck region^(1)^. Feeding requires mastication and swallowing and is related to maintaining the person’s nutritional and hydration status; it also has social, cultural, behavioral, and affective importance^(2)^. Communication, in its turn, is used for social interaction; the voice, with its individual characteristics, is responsible for a large portion of the information that is conveyed^(3)^.

There is an approximately 30% prevalence of disorders related to mastication, swallowing, and voice^(4-6)^. Monitoring – either to confirm the diagnosis or follow up behavioral changes inherent to the treatment – is one of the main and more complex challenges in healthcare for these disorders. Wearable technologies can potentially contribute precisely to this context.

Health-monitoring wearable systems include applications installed on mobile devices (smartphones, tablets, smartwatches, and so forth), which collect the user’s data in natural conditions in their activities of daily living^(7)^. Such technologies are already in use in the field of health to monitor vital signs, such as heart rate, arterial pressure, respiratory rate, blood oxygen saturation, and body temperature, helping follow up the changes that take place throughout therapy or over a given period. Other advantages of wearable technologies include quantitative documentation; investigation outside the setting controlled by the evaluator; automated time of data analysis; greater precision, as it is less dependent on the evaluator; and greater feasibility in clinical routine for both individuals and groups, with greater financial availability and feasibility than some traditional examination instruments^(8)^. Moreover, when compared to traditional methods, wearable technologies generate more accessible data, in greater quantity. In short, the use of wearable systems has the advantage of monitoring the person’s behavior in a natural setting, generating large-scale data that help construct predictive health and behavior models^(9)^.

In the case of mastication, monitoring the usual long-term activity pattern of masticatory muscles may furnish data that precisely represent mandibular function and dysfunction in real-life configurations. Various wearable sensor systems for mastication recognition have already been reported, including microphones^(10,11)^ and intra-auricular proximity sensors^(12)^, deformation sensors^(13,14)^, surface electromyography sensors^(15)^, and accelerometers^(10)^. Such devices are known to interfere minimally with spontaneous mastication behaviors^(16)^, which favors more precise assessments of the functional capacities, the management of excessive muscle activity, and control of bruxism and pain. Hence, wearable technologies used with this objective are a consistent advancement regarding the complex configuration, preparation, and conduction of most mastication assessment instruments available^(17,18)^.

High-resolution sensors, especially accelerometers and piezoelectric sensors, have also helped map swallowing and its disorders^(17,19)^. These sensors pick up spectra of vibratory, acoustic, and displacement signals taking place in the neck region^(8)^. Thus, they help screen, detect, measure, and/or monitor isolated parameters, such as the displacement of structures^(20,21)^ and coordination between swallowing and other functions (e.g., breathing)^(19,22)^, with sensors whose signals are synchronized within a data acquisition system^(8)^. This approach has been encouraging the development of increasingly accessible devices to analyze and monitor real-time swallowing in everyday situations, particularly during meals. Studies point out that wearable technologies generate algorithms with optimal measurement properties to classify individuals regarding their swallowing conditions^(20,23-26)^. There are promising records of machine learning methods being used, such as Deep Neural Networks^(20,23,24,27)^, Support Vector Machines^(28)^, and Linear Discriminant Analysis^(29)^. The use of big data to train these systems will make it possible to define increasingly robust and reliable deep learning models to automatically analyze swallowing parameters.

As for voice, most disorders are caused by abusive vocal behaviors in the activities of daily living. In general, dysphonic patients present with estimates lower than the actual vocal demands in clinical assessment, considering that voice use patterns are automatic and habituated, and people are seldom aware of them^(30)^. In this regard, although clinical voice assessment seeks to map voice production by eliciting various tasks to find the laryngeal dynamics, using wearable technologies with an accelerometer and microphone in the neck region has given promising results and clarified important clinical issues, from assessment to voice rehabilitation^(31)^. These technologies provide measures such as time, cycle, and distance doses; acoustic measures; and aerodynamic measure estimates. The Daily Phonotrauma Index, for instance, is obtained from data collected with wearable technologies; its accuracy for discriminating patients with phonotraumatic lesions from healthy individuals is higher than 85%^(32)^. Furthermore, in the field of voice, wearable technologies can help implement changes in vocal behavior through biofeedback^(33)^. They make it possible to monitor patients in real time, inform them when they have abusive vocal behaviors, and maximize motor learning by reinforcing the patient’s necessary adjustments and calibration. In short, wearable technologies in the field of voice help understand the complex relationship between voice needs and the response to such needs^(34)^.

We would like to conclude our considerations by highlighting that wearable devices can continuously, comprehensively, and simultaneously monitor many signals of functions related to feeding and communication. They generate a large amount of data with the potential to improve the basis of knowledge for decision-making through computer systems that help construct predictive health and behavior models. Patients with difficulties transferring to their everyday life the adaptive or compensatory behavior patterns they learned in healthcare particularly benefit from using these resources. Hence, wearable technologies are an advancement for health services. However, some issues still pose a great challenge, such as concerns with the patient’s privacy, system interoperability, Internet access, and handling a large amount of data per patient. Hopefully, the consolidation of scientific evidence will enable the implementation of wearable technology systems in everyday life to clinically monitor patients.
