# Supplementary material for: Wearable technology use for the analysis and monitoring of functions related to feeding and communication
Source: Codas. 2022 Jul 22;34(5):e20210278. doi: 10.1590/2317-1782/20212021278en (PMC9886183; doi:10.1590/2317-1782/20212021278en)
Supplement: Translation

# Translation: O uso de tecnologias vestíveis para análise e monitoramento de funções relacionadas à alimentação e comunicação

**DOI:** 10.1590/2317-1782/20212021278pt

**Authors:** Bianca Oliveira Ismael da Costa, Alana Moura Xavier Dantas, Liliane dos Santos Machado, Hilton Justino da Silva, Leandro Pernambuco, Leonardo Wanderley Lopes

**Affiliations:** 1 Programa de Pós-graduação em Modelos de Decisão e Saúde, Universidade Federal da Paraíba – UFPB - João Pessoa (PB), Brasil.; 2 Programa de Pós-graduação em Odontologia, Cidade Universitária, Universidade Federal de Pernambuco – UFPE - Recife (PE), Brasil.

**Keywords:** Mastigação, Deglutição, Voz, Dispositivos Eletrônicos Vestíveis, Tecnologia Biomédica

Prezadas editoras,

O uso de dispositivos e sistemas baseados em tecnologias vestíveis tem se revelado uma alternativa contemporânea para superar desafios relacionados à análise e monitoramento de funções relacionadas à alimentação e comunicação. Diante disso, esta carta tem como objetivo comentar sobre esse cenário nas áreas de mastigação, deglutição e voz.

Alimentação e comunicação são atividades indispensáveis à sobrevivência humana que possuem em comum o fato de estarem ligadas a aspectos sociais e emocionais e serem dependentes de ações fisiológicas que ocorrem na região crânio-orocervical^(1)^. O ato de se alimentar acontece por meio da mastigação e deglutição e está relacionado à manutenção do estado nutricional e hídrico, além de carregar significado social, cultural, comportamental e afetivo^(2)^. Já a comunicação é utilizada para a interação social, sendo a voz responsável por grande parte das informações transmitidas, transparecendo características individuais^(3)^.

A prevalência de distúrbios relacionados à mastigação, deglutição e voz é de aproximadamente 30%^(4-6)^. Dentre os principais e mais complexos desafios na atenção a esses distúrbios está o monitoramento, seja com finalidade de apoio ao diagnóstico ou no acompanhamento das mudanças comportamentais inerentes ao tratamento. É justamente nesse contexto que se vislumbra a potencial contribuição do uso de tecnologias vestíveis.

Os sistemas vestíveis para monitoramento da saúde incluem aplicativos instalados em dispositivos móveis (*smartphones, tablets, smartwatches*, entre outros) que permitem coletar dados do usuário em condições naturalísticas durante as atividades de vida diária^(7)^. Tecnologias desse tipo já existentes na área da saúde proporcionam o monitoramento de sinais vitais como frequência cardíaca, pressão arterial, frequência respiratória, saturação de oxigênio no sangue e temperatura corporal, permitindo acompanhar as mudanças que ocorrem ao longo da abordagem terapêutica ou durante um determinado período. Outras vantagens das tecnologias vestíveis incluem: documentação quantitativa; investigação fora do *setting* controlado pelo avaliador; automatização do tempo de análise dos dados; maior precisão por ser menos dependente do avaliador; e maior viabilidade para a rotina clínica no âmbito individual e coletivo, com maior disponibilidade e viabilidade financeira do que alguns exames instrumentais tradicionais^(8)^. Além disso, se comparado aos métodos tradicionais, o conjunto de dados gerados pelas tecnologias vestíveis é maior e de mais fácil acesso. Em síntese, o uso de sistemas vestíveis tem a vantagem de monitorar o comportamento do indivíduo no ambiente natural, gerando dados em larga escala que permitem a construção de modelos preditivos de saúde e comportamento^(9)^.

No caso da mastigação, o monitoramento do padrão de atividade habitual de longo prazo de músculos mastigatórios pode fornecer dados que representam com precisão a função e disfunção mandibular em configurações da vida real. Diferentes sistemas de sensores vestíveis já foram relatados para reconhecimento da mastigação, incluindo microfones^(10,11)^ e sensores de proximidade intra-auriculares^(12)^, sensores de deformação^(13,14)^, sensores de eletromiografia de superfície^(15)^ e acelerômetros^(10)^. Sabe-se que dispositivos deste tipo interferem minimamente no comportamento espontâneo da mastigação^(16)^, o que favorece a avaliação mais precisa das capacidades funcionais, o gerenciamento da atividade muscular excessiva e o controle do bruxismo e dor. Portanto, tecnologias vestíveis usadas com esse objetivo representam um avanço consistente em relação à complexidade de configuração, preparo e execução da maioria dos recursos disponíveis para avaliação instrumental da mastigação^(17,18)^.

O uso de sensores de alta resolução, em especial acelerômetros e sensores piezoelétricos, também tem contribuído para complementar o mapeamento da deglutição e seus distúrbios^(17,19)^. Estes sensores captam espectros de sinais vibratórios, acústicos e de deslocamentos que ocorrem na região do pescoço^(8)^. Portanto, contribuem para rastrear, detectar, mensurar e/ou monitorar parâmetros isolados como o deslocamento de estruturas^(20,21)^ ou a coordenação entre deglutição e outras funções como a respiração^(19,22)^, por exemplo, por meio de sensores com sinais sincronizados pelo mesmo sistema de aquisição de dados^(8)^. Esta abordagem tem incentivado o desenvolvimento de dispositivos de custo cada vez mais acessível para analisar e monitorar a deglutição em tempo real e em situações cotidianas, sobretudo durante as refeições. Estudos apontam que as tecnologias vestíveis permitem gerar algoritmos com propriedades ótimas de medida para classificar indivíduos quanto às condições de deglutição^(20,23-26)^, sendo possível encontrar registros promissores do uso de métodos de aprendizagem de máquina como Redes Neurais Profundas^(20,23,24,27)^, Máquinas de Vetores Suporte (SVMs - *Support Vector Machines*)^(28)^ e Análise Discriminante Linear (LDA - *Linear Discriminant Analysis*)^(29)^. A utilização de grande volume de dados (*big data*) para treinamento desses sistemas possibilitará definir modelos de aprendizagem profunda cada vez mais robustos e confiáveis para realizar análise automática de parâmetros de deglutição.

Quanto à voz, a maioria dos distúrbios é ocasionado por comportamento vocal abusivo nas atividades de vida diária dos indivíduos. De maneira geral, os pacientes disfônicos trazem estimativas inferiores às reais demandas vocais durante a avaliação clínica, considerando-se que os padrões de uso de voz são automáticos e habituados, e que raramente estão no nível consciente^(30)^. Nesse sentido, embora a avaliação vocal no contexto clínico busque mapear a produção vocal eliciando várias tarefas para dar conta da dinâmica laríngea, o uso de tecnologias vestíveis com acelerômetro e microfone na região do pescoço tem apresentado resultados promissores e elucidado questões clínicas importantes, desde o processo de avaliação até a reabilitação vocal propriamente dita^(31)^. Por meio do uso dessas tecnologias é possível extrair medidas como dose de tempo, dose de ciclo, dose de distância, medidas acústicas e estimativas das medidas aerodinâmicas. O índice de fonotrauma diário (*Daily Phonotrauma Index – DPI*), por exemplo, é obtido pela coleta de dados com tecnologias vestíveis e possui acurácia superior a 85% para discriminar pacientes com lesão fonotraumática e indivíduos saudáveis^(32)^. Adicionalmente, na área de voz, as tecnologias vestíveis podem ser utilizadas para dar suporte à implementação de mudanças no comportamento vocal do paciente por meio de *biofeedback* ^(33)^. Elas permitem monitorar o paciente em tempo real, conscientizar sobre os períodos de comportamento vocal abusivo e maximizar a aprendizagem motora com reforço dos ajustes necessários e calibração do paciente. Em síntese, o uso das tecnologias vestíveis na área de voz permite compreender a complexa relação entre demanda vocal e resposta à demanda^(34)^.

Finalizamos nossas considerações salientando que os dispositivos vestíveis são capazes de monitorar de forma contínua, abrangente e simultânea uma grande quantidade de sinais de funções relacionadas à alimentação e comunicação, gerando um número de dados com potencial de melhorar a base de conhecimento para a tomada de decisão por meio de sistemas computacionais que permitem a construção de modelos preditivos de saúde e comportamento. Pacientes com dificuldades em transferir para o cotidiano os padrões de comportamentos adaptativos ou compensatórios aprendidos na clínica serão especialmente beneficiados com o uso desses recursos. Deste modo, as tecnologias vestíveis representam um avanço para os serviços de saúde. Porém, questões como preocupações com a privacidade do paciente, interoperabilidade do sistema, acesso à internet e o manejo de grande volume de dados por paciente ainda representam um desafio. Espera-se que a consolidação das evidências científicas permita a implementação de sistemas de tecnologias vestíveis no cotidiano com vistas à realização do monitoramento clínico.
